# Rate of Correction and All-Cause Mortality in Patients With Severe Hypernatremia

**DOI:** 10.1001/jamanetworkopen.2023.35415

**Published:** 2023-09-28

**Authors:** Eugene Feigin, Libi Feigin, Merav Ingbir, Orit Kliuk Ben-Bassat, Daniel Shepshelovich

**Affiliations:** 1 Internal Medicine Division, Tel Aviv Sourasky Medical Center, Tel Aviv, Israel; 2 Institute of Endocrinology, Metabolism and Hypertension, Tel Aviv Sourasky Medical Center, Tel Aviv, Israel; 3 Faculty of Medicine, Tel Aviv University, Tel Aviv, Israel; 4 Nephrology Department, Tel Aviv Sourasky Medical Center, Tel Aviv, Israel

## Abstract

**Importance:**

Hypernatremia is common among hospitalized patients and is associated with high mortality rates. Current guidelines suggest avoiding fast correction rates but are not supported by robust data.

**Objective:**

To investigate whether there is an association between hypernatremia correction rate and patient survival.

**Design, Setting, and Participants:**

This retrospective cohort study examined data from all patients admitted to the Tel Aviv Medical Center between 2007 and 2021 who were diagnosed with severe hypernatremia (serum sodium ≥155 mmol/L) at admission or during hospitalization. Statistical analysis was performed from April 2022 to August 2023.

**Exposure:**

Patients were grouped as having fast correction rates (>0.5 mmol/L/h) and slow correction rates (≤0.5 mmol/L/h) in accordance with current guidelines.

**Main Outcomes and Measures:**

All-cause 30-day mortality.

**Results:**

A total of 4265 patients were included in this cohort, of which 2621 (61.5%) were men and 343 (8.0%) had fast correction rates; the median (IQR) age at diagnosis was 78 (64-87) years. Slow correction was associated with higher 30-day mortality compared with fast correction (50.7% [1990 of 3922] vs 31.8% [109 of 343]; *P* < .001). These results remained significant after adjusting for demographics (age, gender), Charlson comorbidity index, initial sodium, potassium, and creatinine levels, hospitalization in an ICU, and severe hyperglycemia (adjusted odds ratio [aOR], 2.02 [95% CI, 1.55-2.62]), regardless of whether hypernatremia was hospital acquired (aOR, 2.19 [95% CI, 1.57-3.05]) or documented on admission (aOR, 1.64 [95% CI, 1.06-2.55]). There was a strong negative correlation between absolute sodium correction during the first 24 hours following the initial documentation of severe hypernatremia and 30-day mortality (Pearson correlation coefficient, −0.80 [95% CI, −0.93 to −0.50]; *P* < .001). Median (IQR) hospitalization length was shorter for fast correction vs slow correction rates (5.0 [2.1-14.9] days vs 7.2 [3.5-16.1] days; *P* < .001). Prevalence of neurological complications was comparable for both groups, and none were attributed to fast correction rates of hypernatremia.

**Conclusions and Relevance:**

This cohort study of patients with severe hypernatremia found that rapid correction of hypernatremia was associated with shorter hospitalizations and significantly lower patient mortality without any signs of neurologic complications. These results suggest that physicians should consider the totality of evidence when considering the optimal rates of correction for patients with severe hypernatremia.

## Key Points

**Question:**

Is the rate of correction associated with mortality in patients with severe hypernatremia?

**Findings:**

In this cohort study of 4265 adults with severe hypernatremia, patient survival was strongly correlated with hypernatremia correction rate. There was no evidence of any neurological complications associated with rapid correction of severe hypernatremia.

**Meaning:**

The results suggest that faster correction rates than currently recommended for severe hypernatremia might translate to better patient outcomes.

## Teaser

This cohort study examines whether there is an association between hypernatremia correction rate and patient survival.

## Introduction

Hypernatremia is a common finding in hospitalized patients, with reported incidence 1% to 3%,^1^ and up to 9% in intensive care units (ICUs).^2^ Hypernatremia indicates a deficit in total body water, usually occurring in patients with inadequate access to water or impaired thirst mechanisms such as ICU patients, infants, and older adults.^3^ The clinical presentation of hypernatremia ranges from mild, nonspecific symptoms such as nausea, headache, and lethargy, to severe neurological symptoms such as seizure and coma.^4^ Hypernatremia is associated with higher mortality rates than any other electrolyte abnormality, although this is often attributed to the comorbidities of patients with hypernatremia rather than its direct consequences.^4^

Data regarding the optimal rate of correction of hypernatremia are lacking. Current expert opinion states that hypernatremia correction rate should not exceed 0.5 mmol/L/h or 10 mmol/L/d to avoid the development of cerebral edema and permanent neurological damage.^5,6,7,8^ These complications were documented mainly in neonates.^9,10^ Clinical data supporting the safety and clinical benefits of slow correction of hypernatremia in adults are scarce. Several small studies reported that slow correction of hypernatremia is associated with excess mortality.^7,11^ Faster correction rates of up to 1 millimole per liter per hour have been suggested as appropriate for patients with rapidly developing hypernatremia.^6,8^ A recent study found no association between rate of correction of hypernatremia and clinical outcomes.^5^ Whether current guidelines reflect the optimal balance between patient safety and potential harm is unknown.

The current study aimed to elucidate the association between the rate of correction of hypernatremia and patient outcomes. We hypothesized that fast correction rates would be associated with more favorable patient outcomes.

## Methods

This retrospective cohort study was approved by the Tel Aviv Sourasky Medical Center institutional review board (IRB). Informed consent was waived by the IRB according to local policy for anonymized retrospective studies. We followed the Strengthening the Reporting of Observational Studies in Epidemiology (STROBE) reporting guideline.^14^

### Study Population

The study cohort included all adult patients (aged ≥18 years) admitted between June 2007 and November 2021 to the Tel Aviv Sourasky Medical Center, a tertiary university-affiliated acute care hospital, who had severe hypernatremia (serum sodium ≥155mmol/L [to convert to mEq/L, multiply by 1.0]).^2^ Patients with severe hypernatremia at admission were categorized as admission hypernatremia, and those who developed severe hypernatremia during the hospitalization were categorized as hospital-acquired hypernatremia, consistent with previous studies.^5^ For patients with several episodes of hypernatremia during the study period, only the first episode was included in the present study. We excluded patients with no serum sodium values following documentation of severe hypernatremia and patients on dialysis. Patients with initial blood sodium concentrations exceeding a prespecified threshold of 190 mmol/L as well as those with the 0.05% highest and lowest sodium correction rates between 2 consecutive measurements were also excluded from the final study cohort to avoid outliers stemming from measurement errors of blood sodium levels.

### Data Source, Measurements, and Variables

Data were obtained using MDClone, a query tool that provides comprehensive patient-level data of wide-ranging variables in a defined timeframe around an index event.^12^ Extracted data included age, gender, comorbidities included in the Charlson comorbidity index,^13^ and up to 10 consecutive serum sodium measurements following the first documentation of severe hypernatremia. Serum glucose, magnesium, potassium, creatinine, blood urea nitrogen (BUN), bicarbonate, albumin, calcium, and phosphorous concomitant with severe hypernatremia were also extracted. We also collected data regarding diuretic medications potentially related to dehydration, including furosemide, thiazide and thiazide-like diuretics, aldosterone antagonists, and sodium-glucose cotransporter 2 (SGLT-2) inhibitors. Vital status was ascertained through the patient’s health maintenance organization.

In order to identify potential central nervous system complications of an overly rapid correction of severe hypernatremia, we searched for diagnoses of cerebral edema (*International Classification of Diseases, Ninth Revision, Clinical Modification (ICD-9-CM) *code 348.5), altered mental status (*ICD-9-CM* code 780), seizures (*ICD-9-CM* code 780.39), or epilepsy (*ICD-9-CM* code 345.9). The electronic health records of all patients with 1 of these diagnoses were examined by 1 of the authors (E.F.) to ascertain the associations between hypernatremia and central nervous system complications. Complications were deemed related to hypernatremia if the association was clinically plausible to the treating physicians or if no other reasonable explanation was present upon examination of the patient’s medical record by the investigators.

### Correction Rate Calculation and Definitions

For each patient the initial sodium level was considered as the first sodium measurement equal to or exceeding 155 mmol/L. The subsequent 10 consecutive sodium measurements were recorded. The mean correction rate between documentation of severe hypernatremia and the first documented eunatremia (sodium ≤145mmol/L) was calculated for all patients. For patients with no documentation of eunatremia, the mean correction rate up to the last documented sodium level or the 10th sodium measurement following the documentation of severe hypernatremia was calculated. Fast correction rates were defined as those exceeding 0.5 mmol/L/h, while patients with correction rates less than or equal to 0.5 mmol/L/h were grouped as having slow correction rates. The maximal sodium correction rate was calculated for each patient, defined as the fastest correction rate between any 2 consecutive sodium measurements. The sodium correction within the first 24 hours following documentation of hypernatremia was also calculated for each patient using the latest sodium measurement within the first 24 hours from hypernatremia documentation.

### Study End Points

The primary outcome was all-cause mortality within 30 days. Secondary outcomes were defined as mortality within 7 days and 365 days, length of hospitalization, and rate of neurological complications.

### Statistical Analysis

Patients with fast correction rates were compared with patients with slow correction rates for the entire cohort and stratified by admission hypernatremia vs hospital-acquired hypernatremia. Absolute values of sodium correction for the first 24 hours following documentation of severe hypernatremia were also compared between both groups according to prespecified cutoffs of 8, 10, and 12 mmol/L/d. Categorical variables were compared using the Fischer exact test, and continuous variables were compared using the Wilcoxon rank-sum test. Kaplan-Meier survival curves were compared using the MaxCombo test with the following sets of Flemming-Harrington weights ([0,0], [0,1], [1,0], [1,1]).^15^ Logistic regression was used to determine the association between hypernatremia correction rates and mortality and expressed as the odds ratio (OR). The regression was conducted both with and without adjusting for clinically relevant variables, including demographics (age, gender), Charlson comorbidity index, initial sodium, potassium, and creatinine levels, hospitalization in an ICU, and severe hyperglycemia (defined as glucose levels exceeding 500 mmol/L). Only patients with full data for all of the controlled covariates were included in the adjusted OR (aOR) analysis. Correlation between sodium correction within the first 24 hours and mortality was calculated using the Pearson correlation coefficient. A *P* value of .05 was considered statistically significant for all comparisons. To correct for multiple comparisons, we set for each group of results an adjusted significance threshold based on the Bonferroni correction such that *P* for adjustment <.05 / (number of comparisons). The analysis was conducted using Python version 3.9 (Python Software Foundation). The MaxCombo test was run using the nph library in R version 4.2.1. Statistical analysis was performed from April 2022 to August 2023.

## Results

The cohort included a total of 4265 patients with severe hypernatremia; 2621 (61.5%) were men and 343 (8.0%) had fast correction rates; the median (IQR) age at diagnosis was 78 (64-87) years (Table 1). Median (IQR) Charlson comorbidity index was 5 (3-6) points. The median (IQR) sodium level at diagnosis was 157 (155-159) mmol/L. Most patients had laboratory tests compatible with volume contraction (median [IQR] BUN/creatinine ratio, 32.1 [23.7-42.5]) and relatively few were receiving diuretics. The median (IQR) length of stay at the hospital was 7.1 (3.3-16.0) days.

**Table 1.** Characteristics of Patients With Slow vs Fast Sodium Correction Rates

Characteristics	Patients, No. (%)			*P* value^a^
	Total (N = 4265)	Slow correction (≤0.5 mmol/L/h) (n = 3922)	Fast correction (>0.5 mmol/L/h) (n = 343)	
Age, median (IQR), y	78 (64-87)	78 (64-87)	74 (59-85)	<.001
Gender				
Men	2621 (61)	2466 (63)	155 (45)	<.001
Women				
Charlson comorbidity index, median (IQR) [No.]	5 (3-6) [3903]	5 (3-6) [3582]	4 (2-6) [321]	<.001
Patients in ICU	1255 (29.4)	1154 (29.4)	101 (29.4)	>.99
Extreme hyperglycemic, No. (%)	32 (0.8)	27 (0.7)	5 (1.5)	.18
Laboratory values, median (IQR) [No.]				
Sodium, mmol/L	157.0 (155.0-159.0) [4265]	157.0 (155.0-159.0) [3922]	159.0 (157.0-166.0) [343]	<.001
Creatinine, mg/dL	1.35 (0.94-1.97) [4244]	1.38 (0.97-1.99) [3903]	1.02 (0.70-1.62) [341]	<.001
BUN, mg/dL	46.0 (29.0-69.0) [4260]	48.0 (31.0-70.0) [3917]	24.5 (13.0-50.7) [343]	<.001
BUN/Creatinine	32.1 (23.7-42.5) [4240]	32.8 (24.6-43.0) [3899]	23.1 (15.8-35.2) [341]	<.001
Magnesium, mg/dL	2.28 (2.01-2.56) [2437]	2.29 (2.03-2.57) [2225]	2.10 (1.75-2.44) [212]	<.001
Potassium, mmol/L	3.81 (3.40-4.24) [4253]	3.84 (3.42-4.25) [3910]	3.64 (3.00-4.10) [343]	<.001
Bicarbonate, mmol/L	26.3 (21.7-31.1) [1341]	26.7 (22.0-31.5) [1235]	23.0 (19.5-28.0) [106]	<.001
Albumin, mg/dL	29.0 (25.0-32.9) [3125]	29.0 (25.0-32.0) [2854]	30.0 (24.0-34.5) [271]	.005
Calcium, mg/dL	8.1 (7.5-8.6) [2966]	8.1 (7.5-8.6) [2720]	7.8 (6.6-8.6) [246]	<.001
Phosphorous, mg/dL	3.22 (2.47-4.08) [2858]	3.24 (2.47-4.08) [2623]	3.03 (2.33-4.06) [235]	.19
Medications				
Fusid	659 (15.4)	603 (15.4)	56.0 (16.3)	.64
Aldactone	100 (2.3)	92 (2.3)	8.0 (2.3)	>.99
Thiazide	121 (2.8)	118 (3.0)	3.0 (0.9)	.02
SGLT2 inhibitors	6 (0.1)	5 (0.1)	1.0 (0.3)	.39
Length of stay, median (IQR), d	7.1 (3.3-16.0)	7.2 (3.5-16.1)	5.0 (2.1-14.9)	<.001
7-d mortality	988 (23)	915 (23)	73 (21)	.42
30-d mortality	2099 (49)	1990 (51)	109 (32)	<.001
1-y mortality	3045 (71)	2867 (73)	178 (52)	<.001

Abbreviations: BUN, blood urea nitrogen; ICU, intensive care unit; SGLT2, sodium-glucose cotransporter 2.

SI conversion factors: To convert sodium to mEq/L, multiply by 1.0; creatinine to μmol/L, multiply by 88.4; BUN to mmol/L, multiply by 0.357; magnesium to mmol/L, multiply by 0.4114; potassium to mEq/L, multiply by 1; bicarbonate to mEq/L, multiply by 1; albumin to g/dL, multiply by 0.001; calcium to mmol/L, multiply by 0.25; phosphorous to mmol/L, multiply by 0.323.

^a^ Threshold for statistical significance following adjustment for multiple comparisons is *P* < .002.

Median (IQR) time to correction of hypernatremia was 96 (51-170) hours (Table 2). The median (IQR) overall correction rate was 0.10 (0.04-0.20) mmol/L/h, and median (IQR) maximal correction rate was 0.31 (0.18-0.62) mmol/L/h. The median (IQR) correction rate during the first 24 hours was 2.50 (0.00-5.43) mmol/L. Most patients were corrected according to current guidelines, with only 601 patients (14.1%) corrected by more than 8 mmol/L during the first 24 hours following documentation of severe hypernatremia, 433 (10.1%) corrected by more than 10 mmol/L, and 315 (7.4%) corrected by more than 12 mmol/L.

**Table 2.** Sodium Levels, Correction Rates, and Correction Durations in Patients With Slow vs Fast Sodium Correction Rates

Variable	Patients, No. (%)			*P* value^a^
	Total (N = 4265)	Slow correction (≤0.5 mmol/L/h) (n = 3922)	Fast correction (>0.5 mmol/L/h) (n = 343)	
Initial sodium level, median (IQR), mmol/L	157.0 (155.0-159.0)	157.0 (155.0-159.0)	159.0 (157.0-166.0)	<.001
Time to correction, median (IQR), h	96 (51-170)	102 (66-186)	23 (14-28)	<.001
Overall correction rate, median (IQR), mmol/L/h	0.10 (0.04-0.20)	0.08 (0.04-0.17)	0.83 (0.63-1.50)	<.001
Maximal correction rate, median (IQR), mmol/L/h	0.31 (0.18-0.62)	0.29 (0.17-0.52)	1.14 (0.73-2.03)	<.001
Correction during first 24 h, mmol/L/d	2.50 (0.0-5.43)	2.02 (0.00-4.49)	16.00 (12.83-22.09)	<.001
Correction rate >8 mmol/L/first 24 h	601 (14.1)	292 (7.4)	309 (90.1)	<.001
Correction rate >10 mmol/L/first 24 h	433 (10.1)	132 (3.4)	301 (87.8)	<.001
Correction rate >12 mmol/L/first 24 h	315 (7.4)	45 (1.1)	270 (78.7)	<.001

SI conversion rate: To convert sodium to mEq/L, multiply by 1.0.

^a^ Threshold for statistical significance following adjustment for multiple comparisons is *P* < .006.

There were 3922 patients (92.0%) categorized as having slow correction rates (Table 1). Patients with slow correction rates were older than those with fast correction rates (median [IQR] age: 78 [64-87] years vs 74 [59-85] years; *P* < .001) and had higher median (IQR) Charlson comorbidity index (5 [3-6] vs 4 [2-6]; *P* < .001), a difference driven by a higher prevalence of most comorbidities rather than differences in specific conditions (eTable 1 in Supplement 1). Patients with slow correction rates had higher median (IQR) serum creatinine (1.38 [0.97-1.99] mg/dL vs 1.02 (0.70-1.62) mg/dL; *P* < .001) and BUN/creatinine ratio (32.8 [24.6-43.0] vs 23.1 [15.8-35.2]; *P* < .001), probably reflecting more severe volume contraction. There were no clinically meaningful differences in the prevalence of treatment with diuretics between both groups.

Patients with slow correction rates had median (IQR) initial sodium levels of 157 (155-159) mmol/L, lower than those with fast correction rates (159 [157-166] mmol/L; *P* < .001) (Table 2). The median (IQR) correction rate of patients with slow correction rates was 0.08 (0.04-0.17) mmol/L/h, compared with 0.83 (0.63-1.50) mmol/L/h for those with fast correction rates (*P* < .001). Median (IQR) maximal correction rates were 0.29 (0.17-0.52) mmol/L/h and 1.14 (0.73-2.03) mmol/L/h. Median (IQR) correction rate during the first 24 hours following documentation of severe hypernatremia was 2.02 (0.00-4.49) mmol/L for those with slow correction rates and 16.00 (12.83-22.09) mmol/L for those with fast correction rates (*P* < .001). Among those with slow correction rates, there were 292 (7.4%) documented with absolute correction rates of more than 8 mmol/L/d during the first 24 hours, 132 (3.4%) with more than mmol/L/d, and 45 (1.1%) with more than 12 mmol/L/d; among those with fast correction rates, there were 309 (90.1%) documented with absolute correction rates of more than 8 mmol/L/d during the first 24 hours, 301 (87.8%) with at least 10 mmol/L/d, and 270 (78.7%) with at least 12 mmol/L/d. Median (IQR) time to eunatremia was significantly shorter for those with fast correction rates (23 [14-28] hours vs 102 [66-186] hours for those with slow correction rates; *P* < .001). Median (IQR) patient length of hospitalization was shorter for those with fast correction rates (5.0 [2.1-14.9] days vs 7.2 [3.5-16.1] days; *P* < .001).

**Figure 1.**
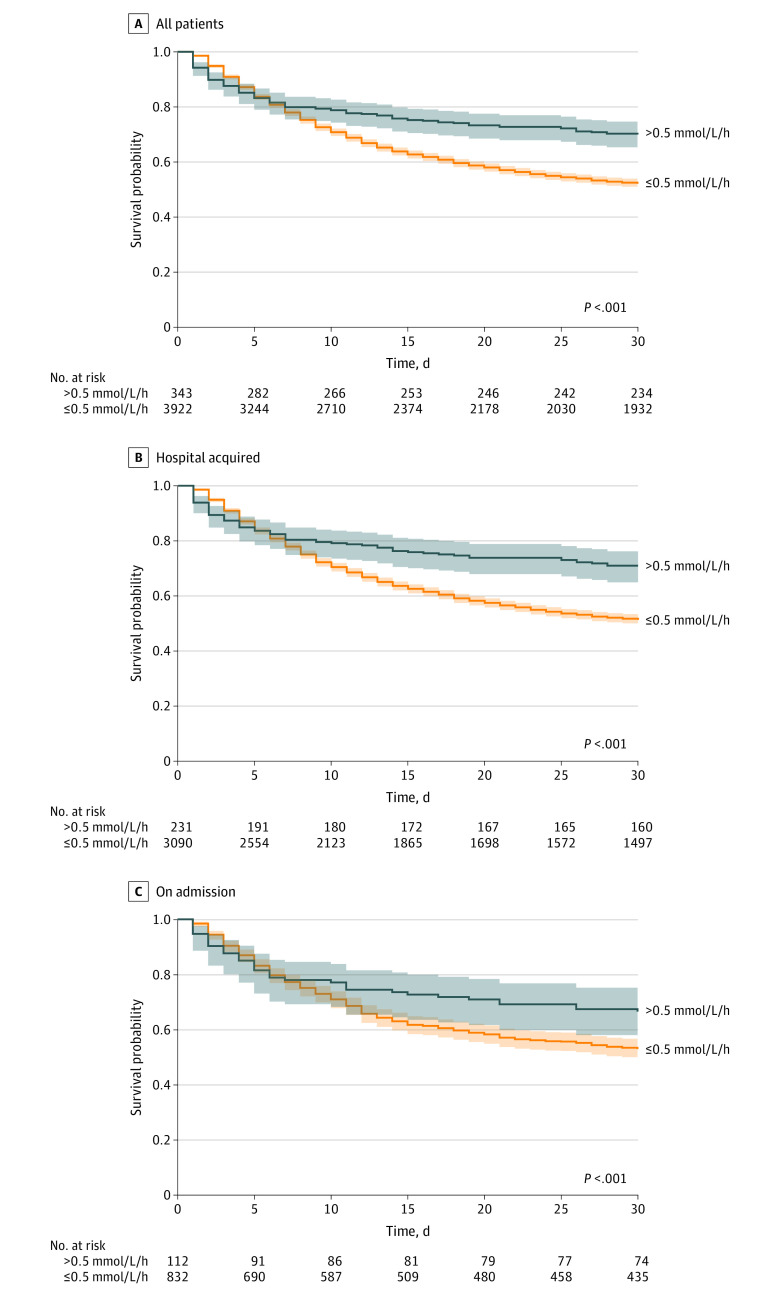
Thirty-Day Survival Curves for Patients With Fast and Slow Sodium Correction Rates

**Figure 2.**
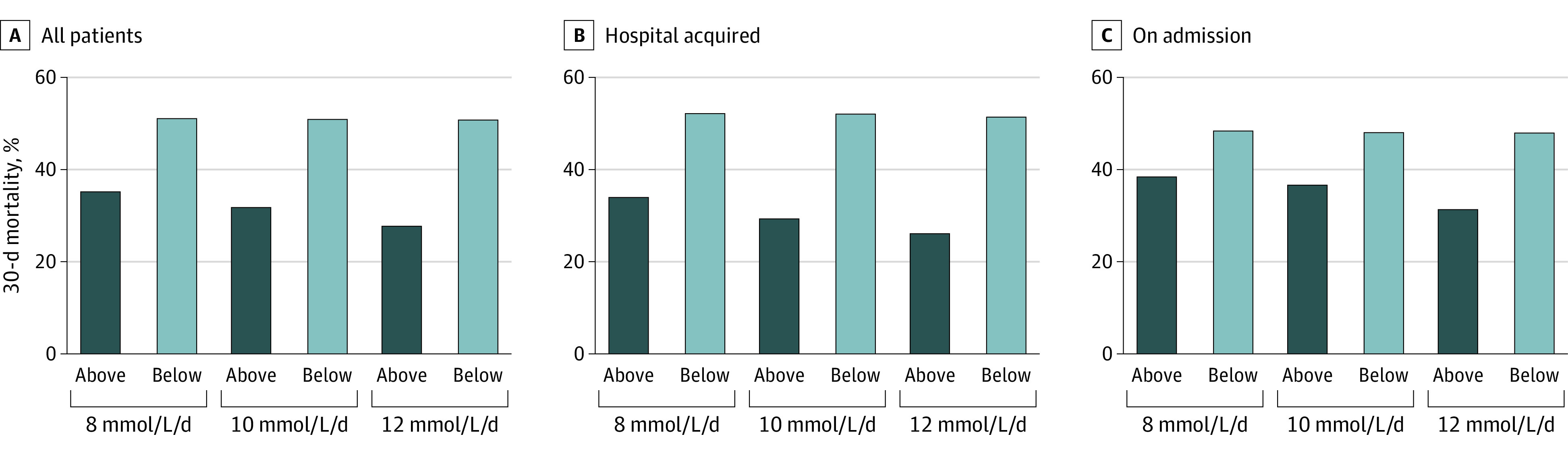
Thirty-Day Mortality According to First 24 Hours Sodium Correction

**Figure 3.**
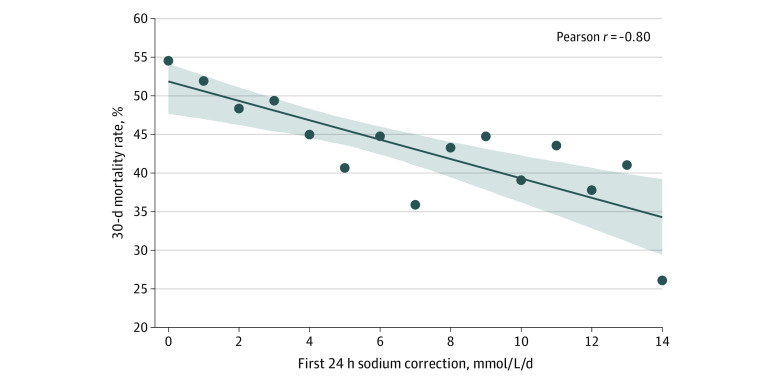
Overall 30-Day Mortality Rate by Absolute Sodium Correction Within First 24 Hours

Both 30-day mortality (50.7% [1990 of 3922] vs 31.8% [109 of 343]; *P* < .001) (Figure 1) and 1-year mortality rates (73.1% [2867 of 3922] vs 51.9% [178 of 343]; *P* < .001) were significantly lower for those with fast correction rates compared with slow correction rates. Short-term prognosis (7-day mortality rates) were comparable between those with slow and fast correction rates (23.3% [915 of 3922] vs 21.3% [73 of 343]; *P* = .42) (Table 1). In multivariate analysis, which included 3891 patients with full data for all variables, correction rates exceeding 0.5 mmol/L/h were associated with increased 30-day survival (aOR, 2.02 [95% CI, 1.55-2.62]). Faster correction of hypernatremia during the first 24 hours was associated with better 30-day survival rates, with a 64.4% (387 of 601) survival rate for patients who corrected more than 8 mmol/L, 67.9% (294 of 433) for patients who corrected more than 10 mmol/L, and 72.1% (227 of 315) for patients who corrected more than 12 mmol/L (Table 2, Figure 2). There was a strong negative correlation between absolute sodium correction during the first 24 hours following the initial documentation of severe hypernatremia and 30-day mortality (Pearson correlation coefficient, −0.80 [95% CI, −0.93 to −0.50]; *P* < .001) (Figure 3). All results remained statistically significant after correcting for multiple comparisons (Table 1 and 2).

There were 3321 patients (77.9%) who had hospital-acquired hypernatremia, and 994 (22.1%) had hypernatremia on admission (eTable 2 in Supplement 1). Patients with hypernatremia on admission were older than those with hypernatremia during hospitalization, with a median (IQR) age of 85 (74-90) years vs 76 (62-85) years (eTable 2 in Supplement 1). They also tended to have more comorbidities (median [IQR] Charlson comorbidity index of 5 [4-6] points vs 4 [3-6] points; *P* < .001). Patients with on admission severe hypernatremia had higher median (IQR) sodium levels (159 [156-163] mmol/L vs 156 [155-158] mmol/L; *P* < .001) and worse kidney function than those with hospital-acquired hypernatremia. Hospitalizations were longer for patients with hospital-acquired hypernatremia. Survival was comparable for both groups in all measured time frames. Mortality rates were significantly lower in rapidly corrected patients both at 30-day (on admission: 33.9% [38 of 112] vs 47.7% [397 of 832]; *P* = .006; hospital acquired: 30.7% [71 of 231] vs 51.6% [1593 of 3090]; *P* < .001) and 1-year (on admission: 59.8% [67 of 112] vs 73.2% [609 of 832]; *P* = .005; hospital acquired: 48.1% [111 of 231] vs 73.1% [2258 of 3090]; *P* < .001) following documentation of severe hypernatremia. These findings remained statistically significant on multivariable analysis (admission hypernatremia: aOR, 1.64 [95% CI, 1.06-2.55]; hospital-acquired hypernatremia: aOR, 2.19 [95% CI, 1.57-3.05]).

There were 12 patients with coded diagnoses of neurological complications: 6 patients with seizures, 5 with changes in consciousness, and 1 with cerebral edema. In all 12 cases, neurological complications were diagnosed and coded prior to initiation of correction of hypernatremia. All 12 cases were in the slow correction rate group. No neurological complications were attributed to rapid rate of correction of hypernatremia by the treating physicians or by the investigators.

## Discussion

Despite the high prevalence of hypernatremia, data guiding optimal correction rates are scarce. To our knowledge, we report on the largest cohort to date of patients with severe hypernatremia. Rapid correction rate of hypernatremia was associated with better overall survival and shorter hospitalizations. We did not identify any evidence supporting an association between rapid rate of correction and patient harm, and no neurological complications were observed in patients with rapid correction. These findings challenge current expert opinion advising slower, controlled correction rates to avoid potentially devastating neurological complications.

Most previous studies of patients with hypernatremia included less than 200 patients.^7,11^ Smaller studies are subject to potentially fragile results leading to coincidental findings, selection bias, and publication bias.^16^ To our knowledge, the largest study to date, including 450 patients, reported no association between hypernatremia correction rate and patient survival.^5^ However, these were patients who were critically ill and hospitalized in ICUs, representing a different patient population. Furthermore, we reported on a considerably larger cohort of patients, making the results more robust.

The currently suggested correction rate of hypernatremia is an extrapolation of the suggested correction rate in neonates, a very different patient population with different physiology, comorbidities, and etiologies for hypernatremia. Faster correction of hypernatremia in adults can theoretically improve patient outcomes in several ways. Faster correction of severe volume contraction and the accompanying acute kidney failure can reduce the risk of potential adverse events, such as electrolyte abnormalities, infections, chronic kidney failure, and cardiovascular complications.^17^ Longer hospitalizations associated with slower correction of hypernatremia are also associated with potential complications such as hospital-acquired infections, falls, delirium and deconditioning, more so in patients with kidney failure, all known to be associated with worse patient outcomes.^18^ Additionally, it is possible that hypernatremia directly causes time-dependent neurological injury, which can be minimized with rapid correction.^19^ This hypothesis, supported by the reported results, should be tested separately, perhaps in an animal model.

Some of the rationale supporting current expert opinion recommending controlled correction of hypernatremia rests on a perceived symmetry with correction of hyponatremia.^20^ While it is appealing to group these 2 common sodium disorders together, published data do not support this approach. The evidence supporting slow correction of hyponatremia is supported by animal studies, numerous case reports and large observational studies.^21^ In contrast, there are no convincing reports of even 1 adult harmed by rapid correction of hypernatremia. From a physiological perspective the risk of neurological complications seems to be much higher with rapid correction of hyponatremia, as the adaptations of brain cells to hypernatremia are much slower than to hyponatremia.^20^ Also, while the risk of complication associated with rapid correction is more common with chronic hyponatremia compared with acute hyponatremia, chronic hypernatremia is considerably less common than acute hypernatremia. Our results were comparable for patients with hypernatremia on admission compared with those who were diagnosed during hospitalization, also supporting the safety of rapid correction of hypernatremia even when there is no clear documentation of an acute condition.

The 30-day mortality was alarmingly high in the reported cohort. This is in line with previous studies of severe hypernatremia.^5,7,11^ The absolute difference in survival between rapidly and slowly corrected patients was very large and remained so after adjustment for potential confounders. Interestingly, the size of the absolute difference in survival was comparable at 30 days and at 1 year following documentation of severe hypernatremia. This might suggest that the different mortality rates do not represent differences in the patient populations unaccounted by the statistical analysis, but rather the results of the treatment strategies for severe hypernatremia, namely the rate of correction. An additional support to this hypothesis is the strong correlation reported between the absolute sodium correction in the first 24 hours and patient survival. This implies that the high mortality rates known to be associated with severe hypernatremia might be partly attributed to lack of aggressive rehydration, which might improve patient outcomes.

### Limitations

This study has several important limitations. First, the retrospective nature of the study increases the risk for bias and confounding. Fast correction of hypernatremia might be a marker of healthier patients, and not directly associated with survival. This seems less likely as results remained statistically significant after adjusting for comorbidities, and as there was no excess mortality in the slow correction rate group between 30 days and 1 year, implying differences in mortality rates are less likely to be attributed to comorbidities and background factors. Unaccounted confounding factors and data entry mistakes might affect the reported results. The substantial discrepancy in sample sizes between the slow and fast correction rate groups, representing class imbalance, might also affect the reported results. Second, many patients were corrected slower than possible under the current guidelines, perhaps due to concerns of overly rapid correction. Of note, the median maximal correction rates in the slow correction rate group are close to currently suggested correction rates for these patients, probably reflecting relatively aggressive initiation of rehydration followed by slower and more careful treatment. Moreover, this slow rate of correction of hypernatremia is similar to rates reported in previous non-ICU studies,^11^ implying slow correction rates might be more common than previously thought and perhaps representing physician concerns of neurological complications of overcorrection. It is possible that faster rates of correction might have translated to better patient outcomes. Third, the study did not include different treatments for hypernatremia, which could potentially influence prognosis. Fourth, our analysis did not include correction of sodium levels for hyperglycemia. However, this is probably not a significant factor for most patients and should be comparable between the 2 studied groups. Also, for the fast correction group, any hyperglycemia would have made true correction rates even higher than currently calculated, underlining the safety of rapid correction. Fifth, our results might not be generalizable to patients with less severe hypernatremia, or for patients in other hospitals. Strengths of our study include the large sample size and the inclusion of all consecutive patients with severe hypernatremia in a predefined time frame.

## Conclusion

In perhaps the largest patient cohort published to date, faster correction of severe hypernatremia than currently suggested was associated with significantly better survival rates. Even very fast correction rates were not associated with adverse neurological events. Health care professionals caring for patients with hypernatremia should take these findings into account when considering the optimal correction rates for their patients.

## Notes

Supplement 1. **eTable 1.** Comorbidities of Patients With Fast vs. Slow Sodium Correction Rates **eTable 2.** Characteristics of Patients With Hospital Acquired Hypernatremia or Hypernatremia Diagnosed On-Admission

Supplement 2.
Data Sharing Statement
