# Supplementary material for: Rate of Correction and All-Cause Mortality in Patients With Severe Hypernatremia
Source: JAMA Netw Open. 2023 Sep 28;6(9):e2335415. doi: 10.1001/jamanetworkopen.2023.35415 (PMC10539989; doi:10.1001/jamanetworkopen.2023.35415)
Supplement: Supplement 1. — eTable 1. Comorbidities of Patients With Fast vs. Slow Sodium Correction Rates eTable 2. Characteristics of Patients With Hospital Acquired Hypernatremia or Hypernatremia Diagnosed On-Admission [file jamanetwopen-e2335415-s001.pdf]

Supplemental Online Content

Feigin E, Feigin L, Ingbir M, Ben-Bassat OK, Shepshelovich D. Rate of correction and all-cause mortality in patients with severe hypernatremia. *JAMA Netw Open.* 2023;6(9):e2335415. doi:10.1001/jamanetworkopen.2023.35415

eTable 1. Comorbidities of Patients With Fast vs. Slow Sodium Correction Rates

eTable 2. Characteristics of Patients With Hospital Acquired Hypernatremia or Hypernatremia Diagnosed On-Admission

This supplemental material has been provided by the authors to give readers additional information about their work.

© 2023 Feigin E et al. *JAMA Network Open.*

eTable 1. Comorbidities of patients with fast vs. slow Sodium correction rates.

	Slow correction ≤0.5 mmol/L/h N = 3922	Fast correction >0.5 mmol/L/h N =343	p
COPD	452 (11.52)	31 (9.04)	0.18
CHF	442 (11.27)	28 (8.16)	0.09
Connective Tissue Disease	72 (1.84)	5 (1.46)	0.83
Dementia	193 (4.92)	11 (3.21)	0.18
Peptic Ulcer Disease	45 (1.15)	3 (0.87)	>0.99
Hemiplegia or Paraplegia	45 (1.15)	3 (0.87)	>0.99
Leukemia	29 (0.74)	3 (0.87)	0.74
Lymphoma	72 (1.84)	8 (2.33)	0.53
Peripheral Vascular Disease	214 (5.46)	12 (3.5)	0.13
CKD	507 (12.93)	36 (10.5)	0.21
AIDS	14 (0.36)	1 (0.29)	>0.99
S/P Cerebrovascular Disease	512 (13.05)	39 (11.37)	0.40
S/P Myocardial Infarction	170 (4.33)	16 (4.66)	0.78
DM "End organ damage"	152 (3.88)	13 (3.79)	>0.99
DM "Uncomplicated"	854 (21.77)	71 (20.7)	0.68
Mild liver disease	108 (2.75)	6 (1.75)	0.38
Moderate liver disease	22 (0.56)	1 (0.29)	>0.99
Local tumor	402 (10.25)	36 (10.5)	0.85
Metastatic tumor	35 (0.89)	1 (0.29)	0.36

Threshold for statistical significance following adjustment for multiple comparisons is $p<0.002$.

© 2023 Feigin E et al. *JAMA Network Open.*

eTable 2. Characteristics of patients with hospital acquired hypernatremia or hypernatremia diagnosed on-admission.

	Total	Hospital acquired hypernatremia	On-admission	p-val
Group size, n (%)	4265	3321 (77.9%)	944 (22.1%)	
Age [years], median (IQR)	78 (64 - 87)	76 (62-85)	85 (74-90)	<0.001
Male gender, n (%)	2621 (61)	2133 (64)	488 (52)	<0.001
Charlson comorbidity index, n (%)	3903	3004	899	
0	308 (8)	244 (8)	64 (7)	0.62
1	213 (5)	181 (6)	32 (4)	0.01
2	294 (8)	266 (9)	28 (3)	<0.001
3≤	3088 (79)	2313 (77)	775 (86)	<0.001
Charlson comorbidity index, median (IQR)	5 (3-6)	4 (3-6)	5 (4-6)	<0.001
Patients in ICU, n (%)	1255 (29.4)	1126 (33.9)	129 (13.7)	<0.001
Extreme hyperglycemic, n (%)	32 (0.8)	13 (0.4)	19 (2.0)	<0.001
Laboratory values, median (IQR), n				
Creatinine [mg/dL]	1.35 (0.94-1.97), n= 4244	1.31 (0.91-1.89), n= 3305	1.53 (1.06-2.26), n= 939	<0.001
BUN [mg/dL]	46.0 (29.0-69.0), n= 4260	44.0 (28.0-66.0), n= 3318	54.0 (33.0-80.0), n= 942	<0.001
BUN/Creatinine	32.1 (23.7-42.5), n= 4240	31.8 (23.4-42.5), n= 3300	32.9 (24.4-42.4), n= 940	0.21
Length of stay (days), median (IQR)	7.1 (3.3 - 16.0)	8.2 (3.9-18.7)	4.7 (2.7-9.3)	<0.001
First sodium [mmol/L], median (IQR)	157.0 (155.0-159.0)	156.0 (155.0-158.0)	159.0 (156.0-163.0)	<0.001
Time to correction [h], median (IQR)	96 (51-170)	97 (54-181)	85 (48-145)	<0.001
Overall correction rate [mmol/L/h], median (IQR)	0.10 (0.04-0.20)	0.08 (0.03-0.18)	0.15 (0.07-0.28)	<0.001
Maximal correction rate [mmol/L/h], median (IQR)	0.31 (0.18-0.62)	0.30 (0.17-0.61)	0.35 (0.20-0.66)	<0.001
Correction during first 24 hours [mmol/L/d]	2.50 (0.0-5.43)	2.02 (0.00-4.75)	4.01 (1.00-8.00)	<0.001
7-day mortality, n (%)	988 (23)	771 (23)	217 (23)	0.90
30-day mortality, n (%)				
Total	2099 (49)	1664 (50)	435 (46)	0.03
≤0.5 mmol/L/h	1990 (51)	1593 (52)	397 (48)	0.051
>0.5 mmol/L/h	109 (32)	71 (31)	38 (34)	0.62
1 year mortality, n (%)	3045 (71)	2369 (71)	676 (72)	0.90

Threshold for statistical significance following adjustment for multiple comparisons is p<0.002.

© 2023 Feigin E et al. *JAMA Network Open.*
