# Supplementary material for: Rate of Correction and All-Cause Mortality in Patients With Severe Hypernatremia
Source: JAMA Netw Open. 2023 Sep 28;6(9):e2335415. doi: 10.1001/jamanetworkopen.2023.35415 (PMC10539989; doi:10.1001/jamanetworkopen.2023.35415)
Supplement: Supplement 2. — Data Sharing Statement [file jamanetwopen-e2335415-s002.pdf]

Data Sharing Statement

Feigin. Rate of Correction and All-Cause Mortality in Patients With Severe Hypernatremia. *JAMA Netw Open*. Published September 28, 2023. doi:10.1001/jamanetworkopen.2023.35415

Data
Data available: Yes
Data types: Deidentified participant data
How to access data: https://github.com/LibiF/Hypernatremia.
When available: With publication

Supporting Documents
Document types: Statistical/analytic code
How to access documents: https://github.com/LibiF/Hypernatremia.
When available: With publication

Additional Information
Who can access the data: researchers whose proposed use of the data has been approved
Types of analyses: Any
Mechanisms of data availability: after approval of a proposal
